# Delayed Graft Function and Its Duration as Predictors of Medium-Term Kidney Transplant Outcomes: A Retrospective Cohort Study from an Eastern European Center

**DOI:** 10.3390/jcm14207240

**Published:** 2025-10-14

**Authors:** Oana Antal, Tudor Moisoiu, Robert Simon, Alina Daciana Elec, Adriana Milena Muntean, Georgeta Horciag, Florina Maria Gabor Harosa, Vlad Pastor, Horia Iuga, Florin Ioan Elec

**Affiliations:** 1 Department of Anesthesia and Intensive Care, Iuliu Hațieganu University of Medicine and Pharmacy, 400012 Cluj-Napoca, Romania; antal.oanna@gmail.com (O.A.); robert.simon@umfcluj.ro (R.S.); 2 Clinical Institute of Urology and Renal Transplantation, 400006 Cluj-Napoca, Romania; tmoisoiu@gmail.com (T.M.); dralinaelec@yahoo.com (A.D.E.); munteana2@yahoo.com (A.M.M.); georgetahorciag@yahoo.com (G.H.); ioan.elec@umfcluj.ro (F.I.E.); 3 Biomed Data Analytics SRL, 400696 Cluj-Napoca, Romania; 4 Doctoral School, Faculty of Medicine, University of Oradea, 410068 Oradea, Romania; 5 Department of Community Medicine, Iuliu Hațieganu University of Medicine and Pharmacy, 400349 Cluj-Napoca, Romania; fgabor@umfcluj.ro; 6 Faculty of Medicine, Iuliu Hațieganu University of Medicine and Pharmacy, 400012 Cluj-Napoca, Romania; iugahoria@gmail.com; 7 Department of Urology, Iuliu Hațieganu University of Medicine and Pharmacy, 400012 Cluj-Napoca, Romania

**Keywords:** kidney transplantation, delayed graft function (DGF), renal replacement therapy (RRT) duration, graft survival, patient survival

## Abstract

**Background/Objectives:** Delayed graft function (DGF) is a major complication after kidney transplantation, affecting graft and patient survival. Although well-studied in Western populations, data from Eastern Europe are limited, and the prognostic significance of DGF severity, particularly renal replacement therapy (RRT) duration, is not well-defined. **Methods:** We conducted a retrospective analysis of 479 adult recipients of brain-dead donor (DBD) kidney transplants at a high-volume transplant center in Romania (2017–2024). DGF was defined as the need for dialysis within seven days’ post-transplant. Baseline characteristics, graft function, and survival outcomes were compared between DGF and non-DGF groups. Kidney function was evaluated using the Estimated Glomerular Filtration Rate (eGFR). Patient and graft survival were assessed using Kaplan–Meier curves and log-rank tests. DGF severity was stratified by RRT duration (≤14 vs. >14 days). **Results:** DGF occurred in 28.8% of patients (adjusted 24%). Those with DGF had a higher Body Mass Index (BMI), greater comorbidity (Charlson Index, Estimated Post-Transplant Survival (EPTS) score), longer pre-transplant dialysis, and higher Kidney Donor Profile Index (KDPI) donor kidneys. DGF was associated with lower graft survival at one, three, and five years and reduced patient survival at three and five years. Longer RRT was associated with progressively worse outcomes, with the poorest prognosis in patients needing >14 days. **Conclusions:** Delayed graft function was significantly associated with reduced graft and patient survival. Prolonged DGF time was found to be predictive for poorer outcomes.

## 1. Introduction

The incidence of chronic kidney disease has demonstrated an upward trajectory in recent decades, with a considerable subset of patients advancing to end-stage renal disease requiring renal replacement therapy [1]. For suitable candidates, kidney transplantation is the preferred therapeutic strategy and confers better survival and quality of life compared with maintenance dialysis [2]. Despite these advantages, postoperative complications may arise, with delayed graft function (DGF) emerging as the complication of greatest clinical importance. DGF is most often defined as the need for dialysis within the first 7 days after transplantation [3].

The development of DGF is multifactorial, with ischemia–reperfusion injury (IRI) as the dominant mechanism. Interruption of blood flow during procurement/cooling/reperfusion leads to acute tubular necrosis [4]. Risk is shaped by donor, recipient, and perioperative features [5].

Short- and long-term complications associated with DGF include immunological, infectious, survival, and health-system components. Immunologically, DGF is associated with greater interstitial fibrosis, tubular atrophy, lower eGFR, and increased graft failure [4,6,7]. The burden of infectious complications is higher, notably, early urinary tract infection and BK viremia, which are probably related to prolonged catheterization, intermittent dialysis exposure, and increased immunosuppression during delayed recovery [8,9]. Long-term graft and patient survival are also affected. However, the duration of DGF represents a critical parameter when assessing its influence on long-term grafts and patient outcomes, as this may better reflect the severity of the underlying acute kidney injury and the organ’s ability to recover [7]. Prolonged DGF duration is known to increase the risk of graft loss in recipients of brain-dead donor (DBD) kidney transplants [10,11].

Although delayed graft function (DGF) is widely recognized as a complication in kidney transplantation, most available evidence comes from multicenter registries or from cohorts in Western Europe and North America, where donor and recipient profiles, healthcare systems, and immunosuppressive protocols differ from those in Eastern Europe. There is limited evidence from high-volume transplant centers in this region assessing the prognostic impact of DGF on both patient and graft survival.

The aim of our study is to assess how DGF affects the short-, medium-, and long-term patient and graft survival in adult kidney transplant recipients of brain-dead donor allografts in a high-volume center of Eastern Europe.

## 2. Materials and Methods

We conducted a retrospective study in kidney transplant recipients treated at the Clinical Institute of Urology and Renal Transplantation in Cluj-Napoca, Romania, between January 2017 and December 2024. This study included patients aged over 18 who received a kidney transplantation from brain-dead donors (DBDs). Exclusion criteria included pediatric recipients, living-donor KTs, and patients with incomplete medical records.

Patients were assessed for delayed graft function (DGF), defined as the need for dialysis within the first seven days after transplantation. Graft function was evaluated using the Estimated Glomerular Filtration Rate (eGFR) recorded in patients’ notes. Graft and patient survival were analyzed with Kaplan–Meier survival curves and compared with the log-rank test.

Intraoperative induction immunosuppression included Basiliximab and methylprednisolone. At the time of graft reperfusion, a 100 mg bolus of furosemide was administered. Fluid management was conducted using normal saline. Blood transfusions were withheld unless hemoglobin levels dropped below 70 g/L.

Anesthesia was induced intravenously with fentanyl, propofol, and either atracurium or rocuronium. Maintenance was achieved using sevoflurane delivered in an oxygen–air mixture with a FiO_2_ of 50% and a fresh gas flow rate exceeding 2 L/min. Additional doses of fentanyl and neuromuscular blocking agents were administered as needed. After induction, a central venous catheter was inserted. Standard ASA monitoring was implemented, including non-invasive blood pressure (NIBP) measurements taken every 10 min.

Surgical time, post-reperfusion time, and any surgical complications in the perioperative period were documented.

The Estimated Post-Transplant Survival score (EPTS) was calculated to offer an additional prognostic assessment following transplantation. This composite scoring system includes four clinical variables: recipient age, duration of dialysis therapy, history of previous solid organ transplantation, and presence of diabetes mellitus.

Graft quality was assessed using the Kidney Donor Profile Index (KDPI), which is a single numerical score that expresses the relative quality of a renal graft. KDPI is derived from the Kidney Donor Risk Index (KDRI), which combines donor characteristics (including age, height, weight, race, history of hypertension and diabetes, cause of death, serum creatinine, HCV status, and donation after circulatory death) using a Cox-model-based formula. Additionally, we determined the predicted 5-year survival for candidates remaining on the transplant waiting list and for those undergoing a KT, using the KDPI-EPTS Benefit Estimator [12].

All grafts were preserved using cold storage.

Maintenance immunosuppression consisted of a calcineurin inhibitor (tacrolimus), mycophenolate mofetil, and corticosteroids, adjusted according to immunological risk and trough levels.

The study protocol received approval from the Ethics Committee of the Clinical Institute of Urology and Renal Transplantation (No. 3/12 May 2022). Due to the retrospective, observational design and anonymized data collection, the requirement for written informed consent was waived.

Statistical analysis was performed using Microsoft Excel 365, IBM SPSS Statistics 27 (IBM Corp., Armonk, New York, NY, USA), and Jamovi version 2.3.28 (Open-Source Project, Sydney, Australia). Continuous variables, when normally distributed with equal variances, were expressed as mean ± standard deviation (SD) and compared with Student’s t-test. If these assumptions were not met, data were reported as median and interquartile range (25th and 75th percentiles) and analyzed using the Mann–Whitney U test. Normality was tested with the Shapiro–Wilk test, and homogeneity of variances was tested with Levene’s test. Survival analysis utilized the log-rank test and Kaplan–Meier curves. The analyzed time points included one month, and one-, three-, and five-year intervals. Two-tailed *p*-values <0.05 were deemed statistically significant.

## 3. Results

During the study period, 607 patients received a kidney transplantation. Following the exclusion of 68 living-donor transplants, 43 pediatric kidney transplants, and 17 cases with incomplete patient records, 479 patients were included in the statistical analysis.

Demographics of the entire study group (*n* = 479) and the two subgroups (i.e., KTx with DGF and non-DGF) are shown in Table 1. The overall incidence of DGF in our cohort was 28.8%. After the exclusion of acute surgical and immunological complications, the adjusted DGF rate was 24%. In total, 21 patients, representing 4.4%, were identified as primary non-function (PNF).

The two groups demonstrated comparable demographic and clinical profiles regarding gender distribution, age, Human Leukocyte Antigen (HLA) mismatches, and antihypertensive medication (Table 1). However, significant differences were found in several patient characteristics, including BMI, Charlson Comorbidity Index, EPTS, duration of hemodialysis, and the prevalence of chronic hypertension and diabetes (Table 1). Donor graft quality, assessed by the KDPI score, also differed significantly between groups.

**Figure 1:**
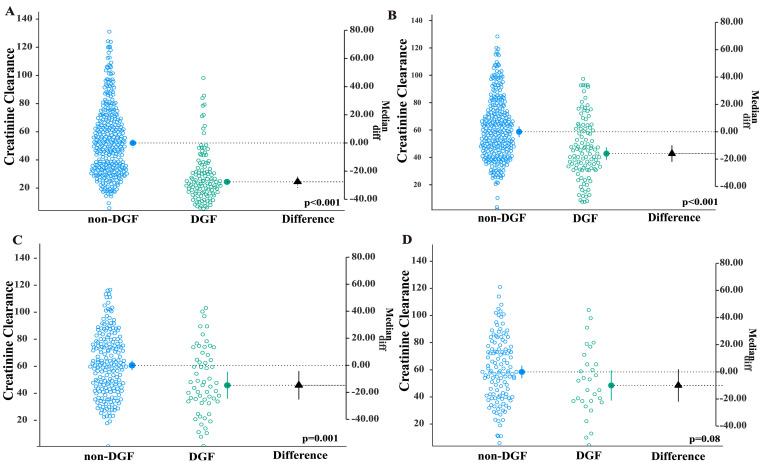
Chronic Kidney Disease Epidemiology Collaboration score (CKD-EPI) at 1 month (**A**), 1 year (**B**), 3 years (**C**), and 5 years (**D**) post-kidney transplantation (KTx).

Estimated Glomerular Filtration Rate, calculated using the Chronic Kidney Disease Epidemiology Collaboration (CKD-EPI) equation, demonstrated statistically significant inter-group differences at 1-month and 1- and 3-year post-transplant time frames, while no significant variation was observed at 5 years following KTx (Figure 1).

**Figure 2:**
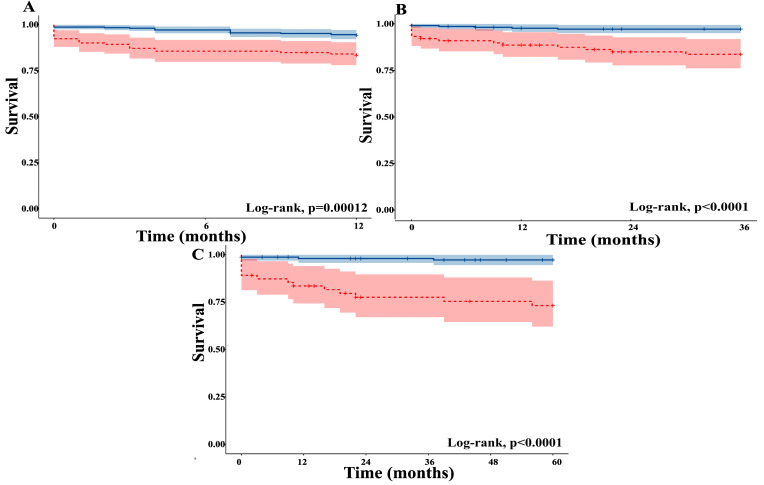
Freedom-from-Death Censored Graft Failure (DCGF) and Freedom-from-Death While Functioning Graft (DWFG) survival at 1 (**A**), 3 (**B**), and 5 years (**C**). The blue part represents the Non DGF group and the red one represents the DGF group.

We analyzed graft and patient survival rates at 1, 3, and 5 years post-transplant, stratified by the presence or absence of DGF. Our findings are displayed in Figure 2 and Table 2. At 1-year post-transplant, twenty-five patients experienced DCGF; an additional seven developed DCGF between years 1 and 3, and four between years 3 and 5. For DWFG, seventeen occurred by one year, an additional seven were out between years 1 and 3, and one between years 3 and 5.

Freedom-from-DCGF and freedom-from-DWFG survival were also analyzed and are plotted in Figure 2.

In order to evaluate whether the prognostic effect of DGF on cause-specific graft loss persisted beyond the early post-transplant period, we performed time-restricted, log-rank analyses. First, we compared DCGS survival between recipients with and without DGF during the first 12 months after transplantation, whereby patients who failed beyond the first year were treated as censored observations. Secondly, to assess prognostic impact beyond the first year, we restricted the cohort to patients who were alive with a functioning graft at 12 months and compared DCGS survival between those who had experienced DGF up to a 3-year post-transplant period. Moreover, we did the same analysis for DGF impact on graft survival up to 5 years after KTx. The log-rank analysis found significant differences among the two groups, in the 1-year, between 1 and 3 years, and 3 to 5 years post-transplant period (*p* < 0.001 in all three time frames).

A statistically significant difference in median hospital length of stay was observed between the two cohorts (*p* < 0.001), with the non-DGF group showing a median duration of 12 days (IQR: 10–14) compared to 26 days (IQR: 19–35) in the DGF group. Patients in the DGF cohort required a median of 10 days of renal replacement therapy (IQR: 5–20).

**Figure 3:**
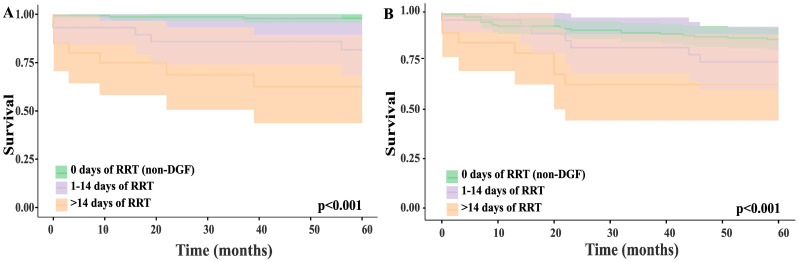
Five-year renal graft (**A**) and patient (**B**) survival rates according to the length of renal replacement therapy (RRT) after transplant.

We conducted a stratified analysis to assess how DGF severity affects graft and patient survival at 1, 3, and 5 years after kidney transplantation, using renal replacement therapy (RRT) duration as an indicator of severity. Initially, patients were divided into four groups: no RRT (343 patients), less than 7 days (38 patients), 7 to 14 days (47 patients), and more than 14 days of RRT (57 patients). Kaplan–Meier survival curves were plotted, and differences between groups were tested with the log-rank test. As no significant differences were found between the <7-day and 7–14-day RRT groups, we combined the categories into three: no RRT (non-DGF patients, 343 patients), RRT lasting 14 days or less (85 patients), and RRT lasting more than 14 days (57 patients). Results are shown in Figure 3.

**Figure 4:**
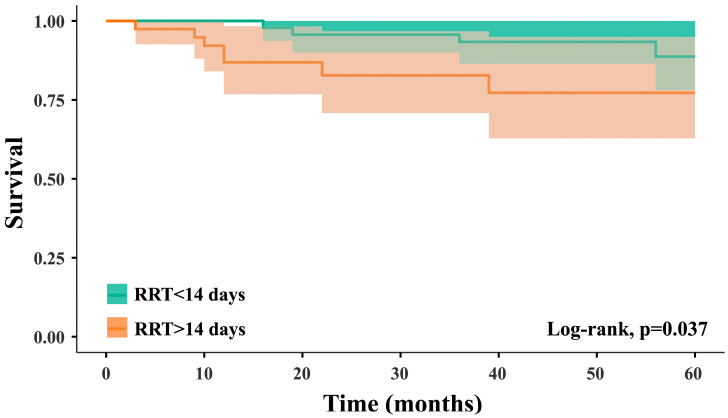
Five-year renal graft survival according to DGF severity.

To address immortal time bias, we performed a Landmark analysis at 60 days post-transplant, conditional on patients being alive with a functioning graft at that time. Comparisons between DGF ≤14 days and >14 days are now based on this approach. We also performed the log-rank test, which was statistically significant, with a *p* = 0.037 (Figure 4).

## 4. Discussion

In this retrospective, single-center analysis of 479 adult kidney transplant recipients from brain-dead donors, the incidence of delayed graft function (DGF) was 28.8%, aligning with rates reported in similar European cohorts [3,13]. DGF was significantly associated with poorer graft survival at 1, 3, and 5 years and with decreased patient survival at 3 and 5 years. Stratification by renal replacement therapy (RRT) duration showed a stepwise decline in outcomes, with the worst results seen in patients requiring RRT for more than 14 days after the kidney transplant.

To our knowledge, this is the first study from Romania and among the very few in Eastern Europe to validate the duration of delayed graft function as a prognostic marker for both graft and patient survival after kidney transplantation.

Our findings support previous meta-analyses showing that DGF negatively impacts long-term graft outcomes [3,13] and is associated with increased morbidity and mortality [14]. The degree of impact in our cohort—particularly the notable decline in 5-year graft survival—may reflect a higher burden of comorbidities, longer pre-transplant dialysis periods, and poorer donor organ quality (higher KDPI) compared to other populations. Importantly, our data suggest that the severity of DGF, measured by RRT duration, provides additional prognostic information beyond the mere presence or absence of DGF, a detail that is less explored in the current literature.

The pathophysiological link between DGF and long-term outcomes is biologically plausible. Ischemia–reperfusion injury during transplantation induces tubular epithelial damage, endothelial dysfunction, and inflammatory activation, which may delay graft recovery, promote alloimmune responses, and accelerate chronic allograft injury. Prolonged RRT likely reflects more severe injury and impaired regenerative capacity.

This study benefits from a relatively large, homogenous DBD-only cohort and detailed perioperative and follow-up data. The use of RRT duration as a surrogate for DGF severity offers a refined risk stratification approach, with potential clinical utility for tailoring post-transplant monitoring and intervention intensity.

We acknowledge several key limitations in our study. First, since it is retrospective, our analysis relies on the accuracy and completeness of existing medical records, which can vary and may lead to information bias. Second, the data originate from a single center, where surgical protocols, perioperative care, and donor selection criteria are specific to our institution. This means that the findings may not fully represent practices or outcomes in other settings but can be representative of Romania.

Notably, the length of renal replacement therapy proved to be a strong indicator of DGF severity, with extended DGF (>14 days) carrying the highest risk of graft loss and mortality. These results highlight the importance of targeted perioperative strategies to reduce ischemia–reperfusion injury, optimize hemodynamic management, and improve donor–recipient matching. Additionally, including DGF severity in prognostic models could improve risk stratification and support personalized follow-up.

Looking ahead, multicenter prospective studies with standardized definitions and thorough adjustment for confounding factors are vital to verify these findings. It will also be important to investigate how DGF interacts with acute rejection and chronic allograft injury, using histology, biomarkers, and imaging to gain a better understanding of the mechanisms involved.

## 5. Conclusions

Delayed graft function was significantly associated with reduced graft and patient survival. Prolonged DGF time was found to be predictive of poorer outcomes.

## Figures and Tables

**Table 1:** Baseline characteristics of all patients, stratified by delayed graft function (DGF) status.

	All Patients	Non-DGF	DGF	*p* Value	RRT < 14 Days	RRT > 14 Days	*p* Value
	(*n* = 479)	(*n* = 337)	(*n* = 142)		(*n* = 81)	(*n* = 44)	
Gender							
M, *n* (%)	308 (64.3)	222 (65.58)	86 (60.56)	0.64	52 (64.2)	29 (35.80)	0.848
F, *n* (%)	171 (35.7)	115 (34.42)	56 (39.44)		29 (65.90)	15 (34.1)	
Age (years), median (IQR)	41 (55–41)	49 (54–40)	52 (56–42)	0.047	52 (55–43)	47 (56.25–36)	0.381
BMI (kg/m^2^), mean ± SD	26.17 ± 4.31	25.8 ± 4.11	27.3 ± 4.6	<0.001	27.51 ± 2.28	26.76 ± 4.66	0.38
Charlson Comorbidity Index, median (IQR)	3 (4–2)	3 (3–2)	3 (4–2)	<0.001	3 (4–2)	3 (5–2)	0.504
KDPI (%), mean ± SD		58.59 ± 27.29	65.50 ± 24.27	0.016	65.14 ± 24.02	64.2 ± 25.44	0.844
EPTS (%), mean ± SD	34.24 ± 26.73	24 ± 24.05	34 ± 30.3	<0.001	40.63 ± 27.57	45.30 ± 35.26	0.45
Hypertension, Yes, *n* (%)		275 (81.6)	109 (76.76)	0.01	62 (76.54)	32 (72.72)	0.638
Diabetes mellitus, Yes, *n* (%)	38 (7.93)	23 (6.82)	15 (10.56)	0.03	9 (11.11)	5 (13.15)	0.931
Number of hypotensive drugs, median (IQR)	2 (3–1)	2 (3–1)	2 (3–1)	0.26	2 (3–1)	1.5 (3–0)	0.352
Dialysis duration, years, median (IQR)	2 (4–1)	2 (4–0.88)	3 (6–1.6)	<0.001	3 (5–1.5)	3 (7–1.5)	0.361
HLA mismatches, median (IQR)	4 (5–3)	4 (5–3)	4 (5–4)	0.07	4 (5–4)	5 (5–4)	0.229
Estimated survival on list, (months), median (IQR)	69.2 (78.2–59.2)	71.4 (79.7–60.6)	65 (75.9–53.8)	0.001	64.2 ± 13.3	63.08 ± 17.81	0.701
Estimated survival if transplant, (months), median (IQR)	89.2 (93.6–80.9)	90.2 (94.1–82)	86.3 (92.4–77.6)	0.001	81.44 ± 11.07	86.37 ± 14.38	0.326

Abbreviations: *n*, number; DGF, delayed graft function; RRT, renal replacement therapy; SD, standard deviation; IQR, interquartile range; EPTS, Estimated Post-Transplant Survival Score; HLA, Human Leukocyte Antigen.

**Table 2:** Patient and graft survival rates in the non-DGF and DGF groups.

	Total	Non-DGF	DGF	*p*
1-year graft survival proportions, Nfunctional/Ntotal, (%)	410/443 (92.5)	303/323 (93.8)	107/120 (89.16)	*p* = 0.033
1-year death-censored graft survival proportions, Nfunctional/Ntotal, (%)	429/443 (96.8)	318/323 (98.5)	111/120 (92.5)	*p* < 0.001
1-year patient survival proportions, Nsurvivors/Ntotal (%)	424/444 (95.5)	309/323 (95.7)	115/121 (95.0)	*p* = 0.728
3-year graft survival proportions, Nfunctional/Ntotal, (%)	263/301 (87.37)	203/224 (90.6)	60/77 (77.9)	*p* < 0.001
3-year death-censored graft survival proportions, Nfunctional/Ntotal, (%)	286/301 (95)	220/224 (98.2)	66/77 (85.7)	*p* < 0.001
3-year patient survival proportions, Nsurvivors/Ntotal (%)	268/302 (88.7)	205/224 (91.5)	63/78 (80.8)	*p* = 0.008
5-year graft survival proportions, Nfunctional/Ntotal, (%)	157/200 (78.5)	127/150 (84.66)	30/50 (60.0)	*p* < 0.001
5-year death-censored graft survival proportions, Nfunctional/Ntotal, (%)	184/200 (92)	147/150 (98.0)	37/50 (74.0)	*p* < 0.001
5-year patient survival proportions, Nsurvivors/Ntotal (%)	165/200 (82.5)	130/150 (86.7)	35/50 (70.0)	*p* = 0.004

Abbreviations: Nsurvivors, number of survivors; Ntotal, number of total patients or grafts; Nfunctional, number of functional grafts.

## Data Availability

The data presented in this study are available on request from the corresponding author due to privacy or ethical restrictions.

## References

[1] Hill N.R., Fatoba S.T., Oke J.L., Hirst J.A., O’Callaghan C.A., Lasserson D.S., Hobbs F.D.R. (2016). Global Prevalence of Chronic Kidney Disease—A Systematic Review and Meta-Analysis. PLoS ONE.

[2] Tonelli M., Wiebe N., Knoll G., Bello A., Browne S., Jadhav D., Klarenbach S., Gill J. (2011). Systematic Review: Kidney Transplantation Compared with Dialysis in Clinically Relevant Outcomes. Am. J. Transplant..

[3] Yarlagadda S.G., Coca S.G., Formica R.N., Poggio E.D., Parikh C.R. (2009). Association between Delayed Graft Function and Al-lograft and Patient Survival: A Systematic Review and Meta-Analysis. Nephrol. Dial. Transplant..

[4] Schröppel B., Legendre C. (2014). Delayed Kidney Graft Function: From Mechanism to Translation. Kidney Int..

[5] Yao Z., Kuang M., Li Z. (2025). Risk Factors for Delayed Graft Function in Patients with Kidney Transplantation: A Systematic Review and Meta-Analysis. BMJ Open.

[6] Siedlecki A., Irish W., Brennan D.C. (2011). Delayed Graft Function in the Kidney Transplant. Am. J. Transplant..

[7] Phillips B.L., Ibrahim M., Greenhall G.H.B., Mumford L., Dorling A., Callaghan C.J. (2021). Effect of Delayed Graft Function on Longer-Term Outcomes after Kidney Transplantation from Donation after Circulatory Death Donors in the United Kingdom: A National Cohort Study. Am. J. Transplant..

[8] Sharif A., Borrows R. (2013). Delayed Graft Function After Kidney Transplantation: The Clinical Perspective. Am. J. Kidney Dis..

[9] Gras J., Le Flécher A., Dupont A., Vérine J., Amara A., Delaugerre C., Molina J.M., Peraldi M.N. (2023). Characteristics, Risk Fac-tors and Outcome of BKV Nephropathy in Kidney Transplant Recipients: A Case–Control Study. BMC Infect. Dis..

[10] Shamali A., Kassimatis T., Phillips B.L., Burton H., Kessaris N., Callaghan C. (2019). Duration of Delayed Graft Function and Out-comes after Kidney Transplantation from Controlled Donation after Circulatory Death Donors: A Retrospective Study. Transpl. Int..

[11] Lim W.H., Johnson D.W., Teixeira-Pinto A., Wong G. (2019). Association Between Duration of Delayed Graft Function, Acute Re-jection, and Allograft Outcome After Deceased Donor Kidney Transplantation. Transplantation.

[12] Bae S., Massie A.B., Thomas A.G., Bahn G., Luo X., Jackson K.R., Ottmann S.E., Brennan D.C., Desai N.M., Coresh J. (2019). Who Can Tolerate a Marginal Kidney? Predicting Survival after Deceased Donor Kidney Transplant by Donor-Recipient Combination. Am. J. Transplant..

[13] Li M.T., Ramakrishnan A., Yu M., Daniel E., Sandra V., Sanichar N., King K.L., Stevens J.S., Husain S.A., Mohan S. (2023). Effects of Delayed Graft Function on Transplant Outcomes: A Meta-Analysis. Transplant. Direct.

[14] Butala N.M., Reese P.P., Doshi M.D., Parikh C.R. (2013). Is Delayed Graft Function Causally Associated with Long-Term Out-comes after Kidney Transplantation? Instrumental Variable Analysis. Transplantation.

